# Medical and Physical Intelligence

**Published:** 1805-03-01

**Authors:** 


### MEDICAL AND PHYSICAL INTELLIGENCE.

[ FOREIGN AND DOMESTIC. ]

The EDITORS feel highly gratified in laying before their Readers, the following very important Communications.

" To Dr. JOHN WALKER,
*Secretary to the Medical Council, Royal Jennerian Society, London.*

" Sir,

" Conceiving that it may correspond with the wishes of the Royal Jennerian Society, I herewith enclose half a dozen copies of the late publication of Government here, as affording no mean prospect of the practicability of carrying vaccination to such an extent as thereby to effect the gradual disappearance of small-pox in British India.

I am Sir,

Your very obedient Servant,

JAMES ANDERSON."

*Fort St. George,*

*August* 13, 1804.

### " GOVERNMENT ADVERTISEMENT.

" PUBLIC DEPARTMENT.

" The Right Honourable the Governor in Council was pleased in an Advertisement, bearing date the 19th of January 1803, to announce to the Native Inhabitants living under the protection of the Government of Fort St. George, the important advantages which were expected to be derived from the practice of vaccination, and the encouragement which His Lordship in Council proposed to afford for the extension of so signal a blessing throughout the territories under this Presidency.

" His Lordship in Council has accordingly had great satisfaction in observing, that the expectation which was formed of the effects of that invaluable discovery, has not been disappointed, as it appears by a statement of patients vaccinated from the month of September 1802, to the month of April 1804, which has been submitted to his Lordship in Council by the Medical Board, that the number amounts to 145,840 persons, without the occurrence of any casualty.

" His Lordship in Council being impressed with confidence, that the statement which has been prepared, cannot fail to produce conviction in the minds of the Native Inhabitants, of the safety and efficacy of the vaccined disease, his Lordship has accordingly directed that it shall be published, and translated into the Native Languages, for the general information of the Inhabitants under this Government."

GENERAL

GENERAL ABSTRACT *of Persons vaccinated by different Medical Gentlemen, and by Native Practitioners at this Presidency, and at the subordinate Stations subject to the Authority of this Government, from the* 1st *of September,* 1802, *to the* 30th *of April,* 1804, *without any Casualty.*

| | *Europeans.* | | *Bramins.* | | *Malabars.* | | *Gentoos.* | | *Mahometans.* | |
|---|---|---|---|---|---|---|---|---|---|---|
| | Males. | Females. | Males. | Females. | Males. | Females. | Males. | Females. | Males. | Females. |
| Presidency, from Nov. 4, 1802, to April 30, 1804. | 19 | 21 | 620 | 370 | 3526 | 4349 | 3584 | 4132 | 1462 | 1327 |
| Centre Division, from March 1, 1803, to April 30. | 34 | 18 | 1064 | 717 | 5070 | 3032 | 6232 | 4296 | 641 | 376 |
| South. Division, from Sept. 1, 1802, to April 30. | 6 | 6 | 231 | 334 | 9363 | 9246 | 7577 | 8507 | 2820 | 1637 |
| North. Division, from Sept. 1, 1802, to Feb. 29. | 6 | 6 | 43 | 32 | 187 | 146 | 698 | 564 | 67 | 36 |
| Mysore Division, from March 1, 1803, to April 30. | 10 | 12 | 408 | 314 | 2571 | 2231 | 970 | 2451 | 1096 | 630 |
| Malabar Province, from March 1, 1803, to Jan. 31. | 18 | 9 | 8 | 0 | 1651 | 428 | 898 | 122 | 693 | 126 |
| Ceded Districts, in September 1803. | 0 | 0 | 0 | 0 | 2 | 4 | 5 | 0 | 12 | 3 |
| Grand Total | 93 | 72 | 2374 | 1767 | 22,370 | 19,436 | 19,964 | 20,072 | 6791 | 4135 |

| | *Half Casts* | | *Portuguese* | | *Pariahs.* | | *Marahatees.* | | *Cannadies.* | | *Rajaputs.* | |
|---|---|---|---|---|---|---|---|---|---|---|---|---|
| | Males. | Females. | Males. | Females. | Males. | Females. | Males. | Females. | Males. | Females. | Males. | Females. |
| Presidency, from Nov. 4, 1802, to April 30, 1804. | 115 | 107 | 117 | 196 | 4012 | 5794 | 0 | 0 | 0 | 0 | 0 | 0 |
| Centre Division, from March 1, 1803, to April 30. | 45 | 7 | 110 | 104 | 2004 | 1507 | 43 | 25 | 159 | 86 | 21 | 14 |
| South. Division, from Sept. 1, 1802, to April 30. | 113 | 17 | 75 | 55 | 9048 | 7084 | 25 | 55 | 0 | 0 | 55 | 45 |
| North. Division, from Sept. 1, 1802, to Feb. 29. | 0 | 0 | 6 | 6 | 150 | 152 | 0 | 0 | 0 | 0 | 0 | 0 |
| Mysore Division, from March 1, 1803, to April 30. | 18 | 20 | 16 | 15 | 3121 | 3008 | 153 | 139 | 6320 | 3757 | 196 | 131 |
| Malabar Province, from March 1, 1803, to Jan. 31. | 0 | 2 | 221 | 111 | 80 | 6 | 0 | 0 | 29 | 16 | 0 | 0 |
| Ceded Districts, in September 1803. | 0 | 0 | 0 | 0 | 4 | 1 | 0 | 0 | 0 | 0 | 0 | 0 |
| Grand Total | 291 | 153 | 605 | 487 | 18,419 | 17,556 | 221 | 219 | 6508 | 3859 | 272 | 190 |

Published by Order of the Right Honourable the GOVERNOR in COUNCIL.

G. BUCHAN,

Chief Secretary to Government.

*Fort St. George, July 28, 1804.*

"To Dr. WALKER.

"Sir,

"The Royal Jennerian Society having some time ago, through you, forwarded to us copies of their Address for the Extermination of Small-pox, with the Plan, Regulations and Instructions for Vaccine Inoculations; we beg leave to return our best thanks for the communication.

"We congratulate you on the happy success of the adoption and dissemination of the invaluable discovery of Dr. Jenner. The exalted and extensive patronage, which your Society enjoys cannot fail to have due influence over the public opinion, and to overcome the many objections and difficulties suggested by prejudice or ignorance, so that the great body of liberal and enlightened men, who compose your Society, will soon have the sincere satisfaction of seeing the beneficial practice which they recommend, extending itself rapidly over every quarter of the globe.

"With the same views, the Managers of the Public Dispensary of Edinburgh established a Vaccine Institution at that charity, in February 1800, for the gratuitous vaccination of the children of the poor; and appointed us surgeons. Since that time we have inoculated nearly four thousand at the institution, besides a great number at their own houses; and although a great proportion of these children have been inoculated with and repeatedly exposed to the variolous contagion, none have been infected by it. On many children, indeed, who had passed regularly through the process of vaccination, eruptions have appeared at different periods afterwards, which by some ignorant people were supposed to be variolous; but which, upon investigation, uniformly turned out to be chicken-pox. Although in some of these cases, the eruptive fever was very severe; sometimes even attended with convulsions; the consequent eruption very numerous, and in a few cases the last of the pustules did not disappear until the fifth or sixth day.

"These cases were repeatedly visited by many medical practitioners of this place, as well as by ourselves, and none of them entertained any doubt of the disease being chicken-pox.

"Besides the number inoculated at the Dispensary, many thousand parcels of vaccine virus have been sent to different parts of the world.

In May 1803, the Managers of the Dispensary circulated an Address to the Clergy of Scotland, a few copies of which we have taken the liberty to enclose.

"By following the plain directions, which we subjoined to the Address, this reverend and patriotic body of men have saved many thousand useful lives by communicating the blessings of vaccination in many remote parts of Scotland, out of the reach of medical aid. Of this fact we are in possession of ample testimony from the reverend gentlemen who have performed the operation themselves, or who have employed some of the most sensible of their parishioners to perform it.

"We

"We were sorry to see the public mind so much agitated of late, by the publication of the cases of Mr. Goldson and others, tending to create doubts of the efficacy of vaccination as a preventive of small-pox; but we are convinced that the multitude of facts which have been brought forward in contradiction to these publications will prevent them from having any permanent effect, even on the most ignorant or prejudiced.

"We are convinced that the time is not far distant when the efforts of the Royal Jennerian Society, and of those who have followed such a glorious example, will be compleatly successful in exterminating the small-pox, so long the scourge of the human race.

"We have the honor to be,

"Sir,

"Your most obedient servants,

"Wm. Farquharson, M. D.

"James Bryce, Surgeon,

"A. Gillespie, Surgeon."

*Edinburgh,*

*January* 25, 1805.

---

A curious fact in Natural History has been observed by Dr. G. Anselmi, Professor of Anatomy at Turin. A snake, called in Italy, Serpe Nero, the Coluber Natrix of Linnæus, is said to be extremely fond of milk, and the country people even pretend that it makes its way into the dairies to gratify its inclination. They even assert, that it is sometimes found entwined round the legs of cows, sucking their teats with such avidity, as to draw blood when their milk is exhausted. Of this fact, which by many had been considered as a popular tale, the Doctor had himself an opportunity of being an eye-witness. "Walking, according to custom," says he, "one morning on the road called the Park, bordered by pastures, containing a great number of sheep and horned cattle, I observed an old but vigorous cow separate from the others, and lowing, with her head raised in the air, her ears erect, and shaking her tail. Surprised at the noise she made, I seated myself on the banks of a stream, and followed her wherever she went with my eyes. After running for some minutes, she suddenly stopped in a sequestered spot, and began to ruminate. Inquisitive to discover the cause, I went to the place. After going into a pond to drink, she came out and waited on the brink for a black snake, which crept from among the brushes, and approaching her, entwined itself round her legs, and began to suck her milk. I observed this phenomenon two successive days, without informing the herdsman. The third day I acquainted him with it, and he told me that for some time the cow kicked at the approach of her calf, and that she could not without difficulty be compelled to suffer it to suck. We took away the snake, which we killed. On the succeeding days, the cow, after in vain waiting for her suckling, ran about the meadow in such a manner that the herdsman was obliged to to shut her up." Dr. Anselmi has discovered, that if the teats of cows be washed with a decoction of tobacco, the ravages of these extraordinary depredators may be effectually prevented.

---

Dr. Blachly gives the following recipe as highly efficacious in the cure of dropsy, by external application. ℞. Saponis, aceti, et spt. vini ana partes æquales. The whole body is to be rubbed with it at bed-time, as long as the patient can bear the application, occasionally giving him brandy or wine. This remedy, joined to the other remedies of dropsies, cures generally in two or three applications; the water disappearing by perspiration. Oedematous legs bound up, with the mixture plentifully rubbed on them, are reduced in size.

---

M. Strauss announces, that a solution of platina, precipitated by ammonia, washed, dried, and exposed to a red heat for half an hour in a covered crucible, may be amalgamated with from five to seven parts of mercury by trituration in a warm mortar. This amalgam may be laid over copper, and the mercury be driven off by heat; a second coating is applied, mixed with chalk, and sprinkled with water, and the plate is again ignited, and afterwards burnished. By this application, copper vessels may be defended from the action of acids.

---

A new composition for preserving inflammable bodies from the effects of fire, has been made known at Hamburgh, by Professor Palmer. It is composed of one part of sulphur, one of red ochre, and six of a solution of copperas.

---

Dr. Batty's Lectures on the Principles and Practice of Midwifery, and on the Diseases of Women and Children, will commence on Monday the 18th of March, at his house in Great Marlborough Street.

---

Dr. John Rush has just published a work, entitled Elements of Life, or the Laws of Vital Matter.

---

Dr. Shadrach Rickeston is engaged in a work on the Means of preserving Health and preventing Diseases, founded principally on an attention to the Non-Naturals in Medicine.

